# Detection of low-level HCV variants in DAA treated patients: comparison amongst three different NGS data analysis protocols

**DOI:** 10.1186/s12985-020-01381-3

**Published:** 2020-07-13

**Authors:** Valeria Caputo, Roberta Antonia Diotti, Enzo Boeri, Hamid Hasson, Michela Sampaolo, Elena Criscuolo, Sabrina Bagaglio, Emanuela Messina, Caterina Uberti-Foppa, Matteo Castelli, Roberto Burioni, Nicasio Mancini, Massimo Clementi, Nicola Clementi

**Affiliations:** 1 Laboratory of Medical Microbiology and Virology at “Vita-Salute” San Raffaele University, 20132 Milan, Italy; 2 Laboratory of Medical Microbiology and Virology, IRCCS San Raffaele Hospital, 20132 Milan, Italy; 3 Department of Infectious Diseases, San Raffaele Hospital, 20132 Milan, Italy

**Keywords:** HCV, DAA failure, RAS detection, NGS

## Abstract

**Background:**

Notwithstanding the efforts of direct-acting antivirals (DAAs) for the treatment of chronically infected hepatitis C virus (HCV) patients, concerns exist regarding the emergence of resistance-associated substitutions (RAS) related to therapy failure. Sanger sequencing is still the reference technique used for the detection of RAS and it detects viral variants present up to 15%, meaning that minority variants are undetectable, using this technique. To date, many studies are focused on the analysis of the impact of HCV low variants using next-generation sequencing (NGS) techniques, but the importance of these minority variants is still debated, and importantly, a common data analysis method is still not defined.

**Methods:**

Serum samples from four patients failing DAAs therapy were collected at baseline and failure, and amplification of NS3, NS5A and NS5B genes was performed on each sample. The genes amplified were sequenced using Sanger and NGS Illumina sequencing and the data generated were analyzed with different approaches. Three different NGS data analysis methods, two homemade in silico pipeline and one commercially available certified user-friendly software, were used to detect low-level variants.

**Results:**

The NGS approach allowed to infer also very-low level virus variants. Moreover, data processing allowed to generate high accuracy data which results in reduction in the error rates for each single sequence polymorphism. The results improved the detection of low-level viral variants in the HCV *quasispecies* of the analyzed patients, and in one patient a low-level RAS related to treatment failure was identified. Importantly, the results obtained from only two out of the three data analysis strategies were in complete agreement in terms of both detection and frequency of RAS.

**Conclusions:**

These results highlight the need to find a robust NGS data analysis method to standardize NGS results for a better comprehension of the clinical role of low-level HCV variants. Based on the extreme importance of data analysis approaches for wet-data interpretation, a detailed description of the used pipelines and further standardization of the in silico analysis could allow increasing diagnostic laboratory networking to unleash true potentials of NGS.

## Introduction

It is estimated that seventy-one million people are infected by hepatitis C virus (HCV), but only 20% of them are aware of their infectious status. Amongst HCV patients receiving a diagnosis, the global therapy coverage is up to 13%. In 2016, this worldwide health problem has been substantially addressed by the World Health Organization (WHO) which set the challenging goal to achieve 65% reduction in mortality and 80% reduction in incidence by 2030 [1].

Up to now, direct-acting antivirals (DAAs), targeting three different viral proteins encoded by NS3, NS5A and NS5B genes, have been used in combination since 2013. The efficacy and safety of these DAAs have significantly improved HCV treatment and made HCV eradication possible for many patients. DAAs treatment can achieve a cure rate of over 95%, meaning that 5% of patients fail the first-round treatment because the selection of resistance-associated substitutions (RAS) which can confer resistance or reduced susceptibility to a certain DAA [2, 3].

Despite the growing understanding of mutations in the development of drug resistance in HCV infection, controversy is still ongoing whether anti-HCV treatment should be guided by the evaluation of mutations correlating with resistance on all three genes possibly undergoing the selective pressure of the therapy. Clinical guidelines from the American Association for the Study of Liver Diseases (AASLD) recommend testing for resistance to NS5A inhibitor, elbasvir, in case of infection with HCV genotype 1a; moreover NS5A RAS testing for RAS Y93H is recommended for genotype 3 infected treatment-naïve patients with cirrhosis [4]. International guidelines are still not clear on testing for the NS3 RAS Q80K in HCV genotype 1a prior to using simeprevir (no longer recommended for therapy [5]), since the effect of this RAS is different for cirrhotic and non-cirrhotic patients: there was a reduction of the sustain virological response at 12 weeks post treatment (SVR12) for cirrhotic patients [6, 7], while no alteration of those without cirrhosis [8]. Current clinical guidelines state that only RAS present at frequencies above 15% need to be considered when choosing antiviral treatments [4] since polymorphisms associated with drug resistance have been also demonstrated by Sanger for higher represented viral variants [9, 10]. However, the detection threshold is still debated as studies showed different results when mutations at a frequency below 15% are investigated [4, 11–15].

**Fig. 1:**
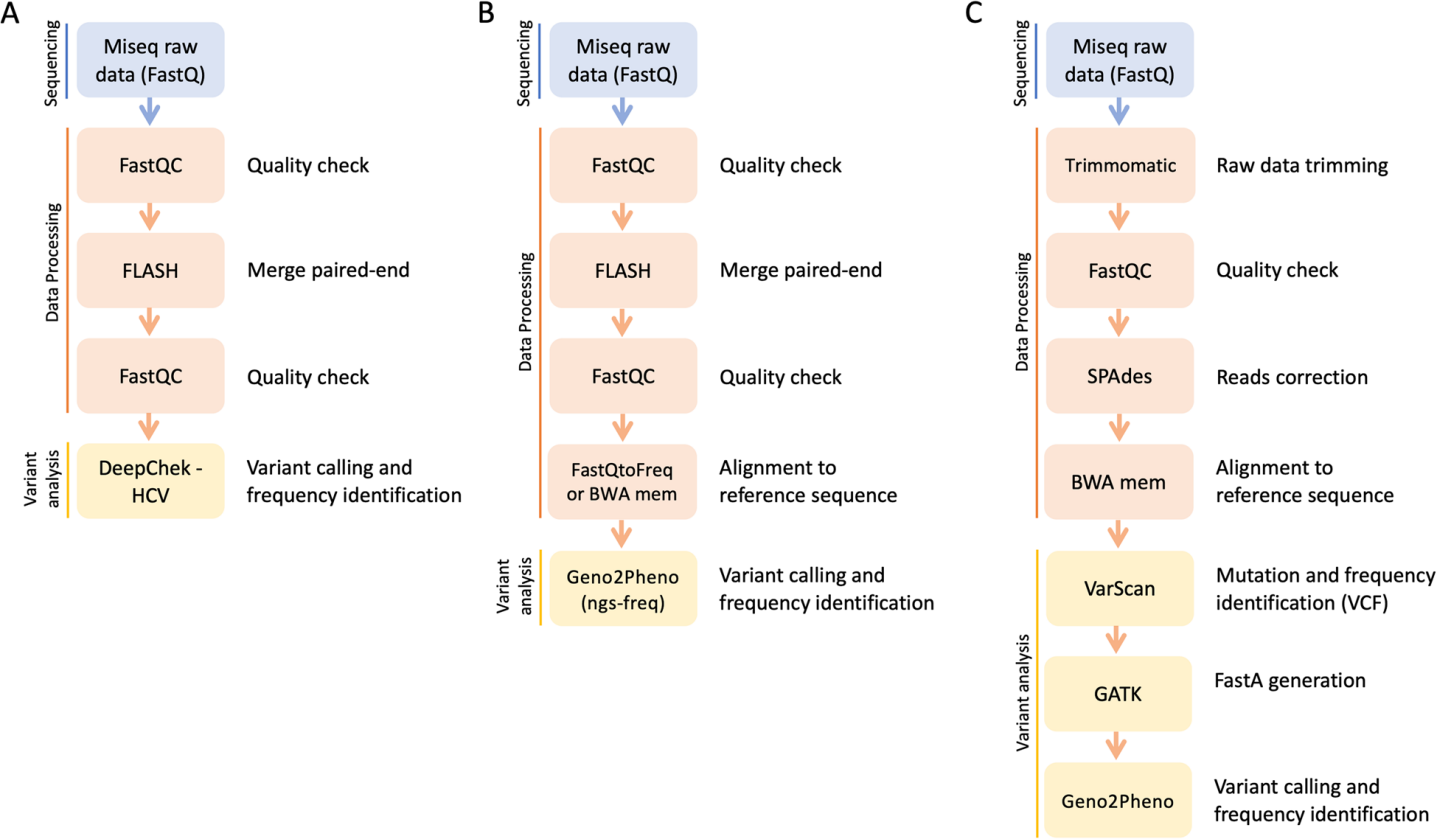
Flowchart of the three methods used for NGS data analysis. **a** first method based on DeepChek® -HCV commercially available certified software; **b** second method based on a homemade raw data analysis coupled with the online informatic tool provided by Geno2Pheno (geno2pheno(ngs-freq)); **c** third method based on a homemade pipeline coupled with VarScan

In the present study, we have tested samples from four HCV patients failing DAAs therapy, by performing Sanger and NGS analysis of the three HCV target genes, NS3, NS5A and NS5B, on plasma samples collected at the beginning and after the failure of DAAs treatment. The data generated by NGS were analyzed using three different strategies (Fig. 1): i) a commercially available certified software ii) a homemade raw data analysis coupled with the online informatic tool provided by Geno2Pheno (geno2pheno(ngs-freq)) and iii) a homemade pipeline.

## Methods

### Patients and clinical history

People included in the study give written consent to collect their data and biological samples. Patient 1 (Pt.1): 52-years-old Caucasian man, HIV infected for 35 years (CDC stage A1). Coinfected with HCV (genotype GT 1b), METAVIR F0, in stable antiretroviral therapy with raltegravir, abacavir and lamivudine, with suppressed plasma viral load and high-level of CD4 (stably > 37%, 650 cells/mL) and naïve to anti-HCV therapy. Patient 2 (Pt.2): 55-years-old Caucasian man, diabetic and HIV infected for 23 years (CDC stage B1). Coinfected with HCV (GT 3a), METAVIR F2, relapse to pegylated interferon plus ribavirin; in stable antiretroviral therapy with darunavir, cobicistat and lamivudine (with suppressed plasma viral load (< 39 cp/mL) and high-level of CD4 (stably > 30%, 600 cells/mL). Patient 3 (Pt.3): 74-years-old Caucasian man, infected with HCV (GT 2c), METAVIR F0, naïve to anti-HCV therapy. Patient 4 (Pt.4): 51-years-old Caucasian man, diabetic and HIV infected for 33 years (CDC stage B2). Coinfected with HCV (GT 4d), METAVIR F4 (cirrhosis CTP A5, MELD 9). Regarding antiretroviral therapy during first-round, the patient was treated with darunavir/ritonavir plus dolutegravir and for drug interactions shift to dolutegravir/rilpivirine plus emtricitabine/tenofovir alafenamide with undetectable HIV RNA (< 39 cp/mL) and high-level of CD4 (stably > 16%, > 400 cells/mL).

### HCV-RNA extraction and amplification

HCV-RNA from collected plasma samples was automatically extracted using QIAamp Viral RNA Mini Kit on QIACube system (QIAGEN, Hilden, Germany) following manufacturers’ instructions. Reverse transcription and subsequent amplification were performed using a pan-genotypic commercially available kit, DeepChek® RT-PCR and Sequencing Kit (ABL SA, Luxembourg, Luxembourg), which led to the amplification of all three DAAs-HCV targeted genes (NS3, NS5A and NS5B). In detail, after a one-step reverse-transcription the amplification for each gene followed a specific and singular nested-PCR protocol, which led to the production of one amplicon of 650 bp for NS3 and of 707 bp for NS5A, and two amplicons for NS5B: a fragment of 693 bp at the 3′ portion of the gene and a fragment of 749 bp at the 5′ portion of the gene. PCR products were analyzed by electrophoresis on 1% agarose gel and size-specific expected bands were purified using QIAquick GEL Extraction Kit (QIAGEN, Hilden, Germany).

### HCV sanger sequencing

Direct sequencing of NS3, NS5A and NS5B was performed by Sanger method on an automatic sequencer ABI PRISM 3100 genetic analyzer DNA Sequencer (Applied Biosystem, Foster City, CA, USA). The samples were prepared using BigDye Terminator v.3.1 cycle sequencing kit (Applied Biosystem, Foster City, CA, USA) followed by a purification step with BigDye XTerminator™ Purification Kit (Applied Biosystem, Foster City, CA, USA). The generated nucleotide sequences were analyzed with SeqScape® Software (ThermoFisher Scientific, Waltham, MA, USA) set at > 25% height of the second peak for IUB assignment and aligned with reference sequence: M58335 for 1b, D50409 for 2c, D17763 for 3a and DQ418786 for 4d (GenBank accession numbers). RASs identification was based on Geno2Pheno algorithm [16].

### HCV next generation sequencing

The same amplicons generated on NS3, NS5A and NS5B, used for Sanger analysis, were sequenced on Illumina MiSeq NGS platform (Illumina, San Diego, CA, USA). Amplicon purification and quantification were performed by Agencourt AMPure XP (Beckman Coulter, Villepinte, France) and Qubit dsDNA Assay Kit (ThermoFisher Scientific, Waltham, MA, USA), respectively. Amplicons from the same patient were pooled and diluted to a final concertation of 0.2 ng/μl and pools were subsequently used for NGS library generation which was performed using Nextera XT DNA Library Prep Kit (Illumina, San Diego, CA, USA). The library generated was then diluted and sequenced with MiSeq Reagent Kit v3 (600-cycles) (Illumina, San Diego, CA, USA) on MiSeq platform. Before starting our sequencing experiment, we used the online ‘Sequencing Coverage Calculator’ (Illumina, San Diego, CA, USA) [17] to estimate the sequencing conditions that allow achieving a depth of coverage (100,000X), where coverage is the number of reads that align to a known reference sequence. This is an essential information because multiple observations per base are needed to obtain a reliable call and, in addition, coverage is also used as a unit for the statistical power of sequencing data [18].

### NGS data analysis

RAS detection and frequency analysis were performed in parallel by using three different approaches: a commercially available certified user-friendly software and two homemade in silico pipeline, one based on the online informatic tool provided by Geno2Pheno (geno2pheno(ngs-freq)) and one based on VarScan (Fig. 1).

For the first two approaches, the quality of raw sequences obtained from MiSeq run was first checked using FastQC (v 0.11.5) (Babraham Bioinformatics), and it was increased using FLASH (v 1.2.11) (Johns Hopkins University Center for Computational Biology), which generated high-quality reads by overlapping paired-end reads using a minimum overlap between R1 and R2 of 20 bp with a maximum of 10% difference.

The approach based on the certified DeepChek®-HCV v2.0 analysis software [19] allowed inferring simultaneously four different HCV databases (Geno2Pheno [16], International Antiviral Society [20], Lontok et al. Hepatology 2015 [21] and Sorbo [22]) and permitted the analysis of minority HCV variant by setting arbitrary thresholds. For our analysis, 1, 5 and 10% thresholds were chosen.

In the second approach, the analysis included the use of fastqToFreq [23] or Burrows-Wheeler Aligner (BWA mem) [24] for the generation of aligned sequences converted as frequencies files, comma-separated values (CSV), that underwent Geno2pheno (ngs-freq) analysis (threshold settings: 1, 5 and 10%) [25].

For the third approach, VarScan (University of Washington in St. Louis) was used for variants identification. The reads were first trimmed and cropped using Trimmomatic [26] and the quality of the sequences was checked using FastQC. The resulting reads were further corrected using SPAdes software (Center for Algorithmic Biotechnology St. Petersburg State University), which correct errors based on specific parameters (reads corrected by BayesHammer) and, successively, reads were aligned with the reference sequence (the same as for Sanger analysis) by BWA mem. Mutations and frequencies were identified by VarScan, which generated variant call format (VCF) files, one for each sample, that were used to generate FastA sequences using Genome Analysis Toolkit (GATK) (Broad Institute). These FastA sequences were finally uploaded and analyzed by Geno2Pheno algorithm [16].

## Results

### Therapeutic DAA regimen and clinical outcome

Pt.1: daclatasvir/pegylated interferon plus ribavirin therapy was started. Virological breakthrough was recorded at week 4, confirmed at week 6 and the therapy was discontinued. Pt.2: relapse at week 12 post-treatment to the first course of therapy included sofosbuvir/velpatasvir plus ribavirin. Pt.3: relapse at week 12 post-treatment with glecaprevir/pibrentasvir. Pt.4: null responder to pegylated interferon plus ribavirin, relapse at week 4 post-treatment with sofosbuvir/ledipasvir plus ribavirin.

### Samples extraction and amplification

Samples were collected at two time-points: before the beginning of the therapy and after therapy failure. All samples underwent reverse-transcription and amplification processes. NS3, NS5A and NS5B were amplified for all samples independently of the therapy regimen adopted and the amplification was correctly performed independently of the genotype for all patients. Only Pt.2 showed problems during NS5B amplification, which resulted in an incomplete amplification of the gene. Only the amplicon at the 5′ portion of the gene at the baseline and, conversely, only the amplicon at the 3′ portion of the gene at the failure were correctly amplified.

### Sanger analysis

Sanger sequencing was followed by Geno2Pheno HCV database analysis [16] (Table 1).

**Table 1:**
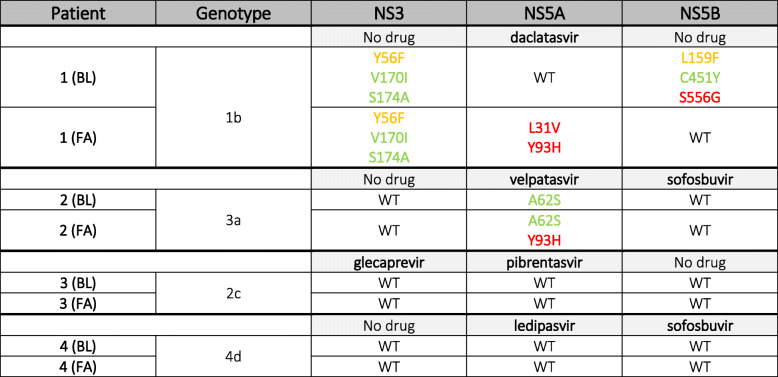
List of mutations detected with Sanger sequencing technique. Mutations in red: RAS; mutations in yellow: reduced susceptibility to DAA(s); mutations in green: mutations on scored positions. BL: baseline; FA: failure; WT: no mutations on scored positions nor described RAS present on HCV databases.

The results obtained from the analysis of patients’ samples baseline revealed that Pt.1 displayed two substitutions on scored position (amino acid substitution not yet described to confer resistance, but present on a sequence position associating with resistance when mutated in other amino acids), V170A and S174A, and one associated with reduced susceptibility to grazoprevir (Y56F) on NS3. The gene NS5A, the only gene undergoing the selective pressure of the subsequent therapy, did not show any mutations, while a substitution on scored position (C451Y), a reduced susceptibility mutation to sofosbuvir (L159F) and a proper RAS to dasabuvir (S556G) were reported on the NS5B. Pt.2 showed a substitution on scored position (A62S) on NS5A gene which underwent the selective pressure of DAA therapy. Pt.3 and Pt.4 did not show any RAS or amino acid substitutions so far described in either one of the genes analyzed at baseline.

Results of the failure revealed that Pt.1 lost all mutations detected at his baseline on NS5B, while maintained the same mutations detected at his baseline on NS3 and acquired two mutations on NS5A: the L31V associated with resistance to daclatasvir and Y93H associated with failure to all inhibitors except for pibrentasvir. Pt.2 showed the same substitution on scored position (A62S) on NS5A, but also a new mutation, Y93H, largely described to be associated with NS5A-inhibitors failure. No described mutations were observed in Pt.3 and Pt.4.

### NGS results: comparison between analysis procedures

NGS sequencing acquired between 839,254-5,470,168 raw reads/sample (Table 2 and 3), which first underwent a quality control check using FastQC to define their Phred quality score (Q score) [27]. To improve the quality of these data, two different approaches were used: FLASH and Trimmomatic software.

**Table 2:** Main features of Illumina MiSeq sequencing Run obtained from Geno2pheno (ngs-freq)

	Total paired read	Combined	Uncombined	% Combined	Number of reads
**1 (BL)**	2,735,084	2,187,422	547,662	79.98%	NS3 28,256
					NS5A 980,338
					NS5B 627,791
**1 (FA)**	419,627	122,250	297,377	29.13%	NS3 4259
					NS5A 44,002
					NS5B 43,337
**2 (BL)**	2,211,307	1,677,988	533,319	75.88%	NS3 783,853
					NS5A 474,463
					NS5B 40,397
**2 (FA)**	1,978,054	1,429,660	548,398	72.28%	NS3 191,206
					NS5A 495,980
					NS5B 386,943
**3 (BL)**	1,978,438	1,288,921	689,517	65.15%	NS3 71,599
					NS5A 151,920
					NS5B 536,113
**3 (FA)**	1,955,249	1,169,929	785,320	59.84%	NS3 22,792
					NS5A 70,599
					NS5B 854,945
**4 (BL)**	1,872,852	1,429,177	443,675	76.31%	NS3 31,485
					NS5A 401,883
					NS5B 643,826
**4 (FA)**	1,915,206	1,382,401	532,805	72.18%	NS3 8880
					NS5A 456,266
					NS5B 650,246

**Table 3:** Main features of Illumina MiSeq sequencing Run obtained after Trimmomatic, SPADES and SAMtools application

	Total reads	Total reads after Trimmomatic	Total reads after SPADES	Number of reads aligned
**1 (BL)**	5,470,168	5,220,204	5,219,176	NS3 88,766
				NS5A 2,952,378
				NS5B 1,722,432
**1 (FA)**	839,254	622,494	619,064	NS3 30,329
				NS5A 232,894
				NS5B 273,387
**2 (BL)**	4,422,614	4,248,300	4,207,974	NS3 2,184,866
				NS5A 1,476,874
				NS5B 136,248
**2 (FA)**	3,956,108	3,726,938	3,677,042	NS3 455,654
				NS5A 1584.345
				NS5B 1,251,540
**3 (BL)**	3,956,876	3,611,990	3,535,350	NS3 244,237
				NS5A 649,235
				NS5B 1,746,295
**3 (FA)**	3,910,498	3,669,880	3,607,316	NS3 80,326
				NS5A 166,688
				NS5B 3,136,304
**4 (BL)**	3,830,412	3,636,298	3,591,446	NS3 30,775
				NS5A 1,416,729
				NS5B 1,925,610
**4 (FA)**	3,745,704	3,590,586	3,555,526	NS3 112,212
				NS5A 1,284,284
				NS5B 1,967,277

FLASH software allowed to reach a Q score ~ Q36 with a consequent decrease in the total reads to 122,250-2,187,422 paired read/sample (Table 2). For the Illumina system, a Q score as 36 implied that the probability of incorrect base calls is among a range of 1:1000–1:10,000 with a range of base call accuracy between 99.9–99.99%. Despite the decrease of the total number of paired reads per gene (4259-980,338; Table 2), each gene was characterized by a high coverage. The sequences obtained from this approach were then analyzed with DeepChek®-HCV v2.0 and geno2pheno(ngs-freq) software.

The second method entailed the use of Trimmomatic which increased the Q score of each sample sequences up to ~ Q37. However, in this case, the quality decreased till ~ Q30 towards the 3′ terminal and the sequences obtained were processed with a homemade pipeline based on VarScan.

The NGS analysis was performed for all patients showing different results.

Regarding Pt.1, NGS data analysis obtained with DeepChek®-HCV v2.0 and geno2pheno(ngs-freq) online platform revealed different results at his baseline: DeepChek®-HCV v2.0 software detected mutation L159F and RAS S556G on NS5B, while geno2pheno(ngs-freq) analysis did not identify RAS S556G. Interestingly, we observed that fastqToFreq tool adopted a reference sequence not covering NS5B full length, excluding 3′-gene portion containing the amino acid position S556. To bypass this major problem, BWA mem tool was used to align raw data to Sanger reference sequence (M58335) in order to create BAM files further converted to CSV files. CSV frequency file was then used to run analysis on geno2pheno(ngs-freq) platform, generating results comparable to DeepChek®-HCV v2.0 analyses. Regarding VarScan results, the analysis correctly detected RAS S556G at Pt.1 baseline, but it did not reveal L159F mutation (Table 4). At Pt.1 failure, NS5B RAS S556G was not detected with all three NGS analysis approaches, but L159F NS5B mutation was identified at a very low-frequency (detected only with DeepChek®-HCV v2.0, 5.56% and geno2pheno(ngs-freq), 5%, methods) (Table 4). Analyses on Pt.1 NS3 and NS5A genes, instead, agreed with all three analysis approaches: we found the mutation Y56F on NS3, while RAS L31V, P58S e Y93H on NS5A gene.

**Table 4:**
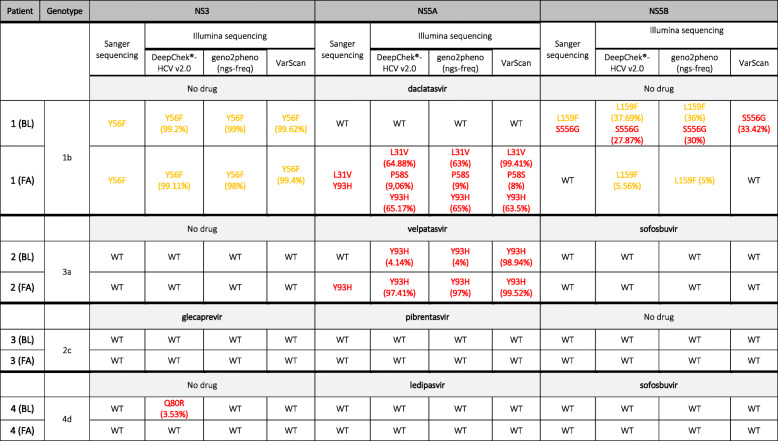
Data comparison between Sanger and Illumina results analyzed with three different approaches. Mutations in red: RAS; mutations in yellow: reduced susceptibility to DAA(s). BL: baseline; FA: failure; WT: no mutations present on HCV databases.

NGS analysis on Pt.2 allowed to detect a low-level viral variant at his baseline: the Y93H RAS on NS5A, which confers full resistance to several DAAs, including velpatasvir (Table 4). Importantly, the frequency of Y93H mutation inferred by DeepChek®-HCV v2.0 and geno2pheno(ngs-freq) was comparable (4.13 and 4%, respectively). On the contrary, VarScan analysis, although detecting the same mutation at the baseline, it identified its frequency at 98.94%. This RAS underwent the selective pressure of velpatasvir (used in combination with sofosbuvir for the treatment of this patient), in fact, as showed at his failure, this RAS was identified at the high frequency with all three analysis approaches (97.41% with DeepChek®-HCV v2.0, 97% with geno2pheno(ngs-freq) and 99.52% with VarScan). Regarding the analysis of the other genes, no mutations were identified at both time points.

Pt.3 *quasispecies* did not show any mutation described on inferred databases [16, 20–22] at any time point and at all tested thresholds (Table 4).

NGS analyses on Pt.4, featuring wildtype *quasispecies* on NS5A and NS5B genes at both tested time points, showed the RAS Q80R on NS3, only at his baseline, which was not targeted by DAAs therapy. In particular, this RAS was observed at a frequency lower than 4% only using Sorbo et al. database available with DeepChek®-HCV v2.0 software [22]. Conversely, the other two approaches, inquiring Geno2Pheno database, did not detect this mutation as RAS (Table 4).

In conclusion, DeepChek®-HCV and geno2pheno(ngs-freq) online platform results confirmed and completed those obtained from Sanger analysis (Table 4), while the results of the analysis with VarScan were not fully coherent.

## Discussion

A great deal of studies is exploring the potentiality and feasibility of NGS sequencing on different virological applications, including the detection of low-level variants associated with drug resistance. This is still an unresolved subject regarding their importance for guiding therapy choices. However, this major problem is preceded by the need to find a common and reliable method for the analysis of NGS generated data that minimizes the error rate during the analysis [28].

This scenario fits perfectly the case of HCV minor variant consideration, and the results of this study draw attention to the need of identifying a consensus system that combined a fine analysis of low-frequency HCV variants and a robust approached that could be used in a diagnostic laboratory and would also help to standardize the results for the correct detection of HCV low-level variants.

In this study, plasma samples of four HCV positive DAA naïve patients were collected before starting the DAAs treatment and at their therapy failure to infer low-level virus mutants within the *quasispecie*s possibly related to therapy failure.

The study was performed using Sanger sequencing, which is still considered the gold standard for the correlation of HCV mutations with therapeutic failure, and the NGS sequencing Illumina system.

Since a key focus was given to the understanding of the role of RAS frequency and the identification of the low-level viral populations in the context of therapy failure using three different NGS data analysis strategies, MiSeq run experimental conditions were tuned to obtain a high number of reads, leading to high coverage of the three genes analyzed. This high coverage allowed a strict analysis that resulted in numerous reads with a Q score (≥ 36) greater than for “traditional” NGS analysis (= 30) and Sanger sequencing (= 20). As reported above, the Q score obtained is an indication of a high accuracy that minimizes possible errors, meaning that low-level HCV variants, still represented by a high number of reads, were detected correctly. Moreover, to identify a possible diagnostic workflow, all sequencing methods used the same amplicons of the three genes codifying for DAAs targets (NS3, NS5A and NS5B) generated by using a commercially available set of primers already used in diagnostic laboratories.

Three NGS raw data analysis strategies were chosen: 1) the Deepchek® -HCV is a commercially available user-friendly and certified software, it allows detecting all mutations at different thresholds consulting simultaneously four different databases; 2) the Geno2Pheno (geno2pheno(ngs-freq)) based pipeline is a free approach, it does not require particular bioinformatics expertise for the generation of the required files and, more importantly, RAS detection is based on online informatic tool provided by commonly used Geno2Pheno. The main drawback of this strategy is that it only detects the mutations associated with full or partial resistance to DAAs and the amino acid variation found on scored position. 3) the last method is a homemade free pipeline which allows, as Deepchek® -HCV, a complete variant calling of every mutation found in the *quasispecies.* This method is based on a widely used tool, VarScan, that is not specific for viral variant calling but for somatic mutation in humans. However, until today, there is no specific free tool for HCV variant calling.

The results obtained from the data analysis illustrate different scenarios based on the software used. The RAS and mutations associated with reduced susceptibility to DAAs detected were coherent with all three analyses protocols except for mutation L159F on NS5B of Pt.1 baseline and failure. This mutation was detected at his baseline at a frequency higher than 20% by Sanger, DeepChek®-HCV v2.0 software and geno2pheno(ngs-freq) analysis. But only VarScan approach did not detect this mutation. Furthermore, the same mutation at Pt.1 failure was observed at a lower frequency only by DeepChek®-HCV v2.0 (5.56%) and geno2pheno(ngs-freq) approaches (5%).

The other relevant difference among the three NGS data analysis systems involved the RAS Y93H on NS5B of Pt.2. This mutation was detected with DeepChek®-HCV v2.0 software and geno2pheno(ngs-freq) and, according to its very low-frequency (4%), Sanger analysis was not able to detect this mutation falling below its threshold. In contrast to these results, the outcome given by VarScan set the frequency level of RAS Y93H at 98,94%. The latter represents an artifact generated by VarScan pipeline, since Sanger approach did not identify this mutation. The discordances observed using VarScan may be due to biases introduced by the script adopted for performing VarScan data processing. It must be underlined that the same script confirmed all mutation frequencies found in the whole analyses.

These discrepancies further stress the need of a well described sequence analysis protocols to be used for diagnostic purposes that couples a user-friendly raw data processing and an HCV specific variant calling tool. In this study, geno2pheno(ngs-freq) protocol well corroborates data obtained with the commercially available certified DeepChek®-HCV v2.0 software, and they integrate also Sanger analysis results; therefore, it could be included amongst reliable tools for data generation and comparison between different research groups.

Notwithstanding the restricted cohort of analyzed patients, our observations further stress the need to reconsider the RAS analysis guidelines to improve DAA management. In fact, studies considering such low-frequency variants often report discordant results. The latest clinical guideline by the AADLS (2018) agreed that the presence of low-frequency RASs may not convey enough resistance to currently available DAA regimens resulting in the development of sustain virological response [4]. Nevertheless, other studies observed the importance of minor variants associated with clinical outcomes [11–15], as we also reported in our work. Therefore, detecting low-level RAS Y93H on the NS5A of Pt.2 at his baseline can be important to further stress the correlations, described by AADLS, between the presence of Y93H at the baseline in patients treated with sofosbuvir/velpatasvir and reduced SVR12 [4]. Moreover, the analysis of very low represented RAS in all genes possibly undergoing selective pressure of DAAs (NS3, NS5A, NS5B) allowed to better characterize mutants’ dynamics of *quasispecie*s independently from the therapeutic regimen. In fact, as observed in our study, mutations possibly related to reduced DAAs susceptibility are present also on genes not undergoing selective drug pressure. At this purpose, also whole-genome sequencing performed at a high depth could be extremely useful when considering possible second-line therapy combinations [12, 29, 30].

Finally, from the in silico part of our pilot study, we evidenced another important issue possibly hampering a diagnostic consensus amongst laboratory data: the identification of a unique reference database to avoid biases due to the interpretation of observed mutations. In fact, as we reported in our work, mutation Q80R on NS3 is considered as RAS only by Sorbo et al. database [22], while for the other databases the same substitution is not associated with confirmed resistance.

## Conclusions

As for other diagnostic fields, the decreasing costs of NGS could certainly allow performing these analyses also in clinical routine to assist in the care of HCV subjects receiving DAA [29, 31]. Given the high rate of DAA therapy success, large cohorts of well stratified samples from DAA-therapy failures are not so common. Therefore, multicentric studies focusing on enrolment of larger cohorts of patients for NGS-based evaluation of minority variants associated with DAA failure would be needed. Moreover, to further investigate the role of clinically relevant minority HCV variants, a defined consensus amongst diagnostic virology laboratories on the use of specific wet and in silico procedures for NGS and data analysis should be desirable.

## Data Availability

Data analysis results are included in the article.

## Abbreviations

AASLD: American Association for the Study of Liver Diseases
BL: Baseline
BWA mem: Burrows-Wheeler Aligner
CSV: Comma-separated values
DAAs: Direct-acting antivirals
FA: Failure
GATK: Genome Analysis Toolkit
GT: Genotype
HCV: Hepatitis C virus
NGS: Next-generation sequencing
Pt: Patient
Q score: Phred quality score
RT-PCR: Reverse transcriptase-polymerase chain reaction
RAS: Resistance-associated mutations
SVR: Sustain virological response
VCF: Variant call format
WHO: World Health Organization
WT: Wildtype
